# Evaluation of Cytotoxicity and Acute Oral Toxicity of Saline Extract and Protein-Rich Fraction from *Moringa oleifera* Lam. Leaves

**DOI:** 10.3390/ph17081045

**Published:** 2024-08-08

**Authors:** Robson Raion de Vasconcelos Alves, Alisson Macário de Oliveira, Gabryella Borges dos Prazeres, Abdênego Rodrigues da Silva, Franciele Florencio Costa, Bárbara Rafaela da Silva Barros, Talita Giselly dos Santos Souza, Luana Cassandra Breintenbach Barroso Coelho, Cristiane Moutinho Lagos de Melo, Magda Rhayanny Assunção Ferreira, Luiz Alberto Lira Soares, Cristiano Aparecido Chagas, Maria Lígia Rodrigues Macedo, Thiago Henrique Napoleão, Mariana Pinheiro Fernandes, Patrícia Maria Guedes Paiva

**Affiliations:** 1 Departamento de Bioquímica, Centro de Biociências, Universidade Federal de Pernambuco, Recife 50670-901, PE, Brazil; robson.raion@ufpe.br (R.R.d.V.A.); alissonmacario@hotmail.com (A.M.d.O.); gabryella.borges@ufpe.br (G.B.d.P.); rodriguesabdenego@gmail.com (A.R.d.S.); lcbbcoelho@gmail.com (L.C.B.B.C.); thiago.napoleao@ufpe.br (T.H.N.); 2 Departamento de Farmácia, Centro de Ciências da Saúde, Universidade Federal de Pernambuco, Recife 50670-901, PE, Brazil; francieli.costa@ufpe.br (F.F.C.); magda.ferreira00@gmail.com (M.R.A.F.); luiz.albertosoares@ufpe.br (L.A.L.S.); 3 Departamento de Antibióticos, Centro de Biociências, Universidade Federal de Pernambuco, Recife 50670-901, PE, Brazil; barbara.sbarros@ufpe.br (B.R.d.S.B.); cristiane.melo@ufpe.br (C.M.L.d.M.); 4 Centro Acadêmico de Vitória, Universidade Federal de Pernambuco, Vitória de Santo Antão 55608-680, PE, Brazil; talitagiselly@hotmail.com (T.G.d.S.S.); cristiano.chagas@ufpe.br (C.A.C.); mariana.fernandes@ufpe.br (M.P.F.); 5 Departamento de Tecnologia de Alimentos e da Saúde, Faculdade de Ciências Farmacêuticas, Alimentos e Nutrição, Universidade Federal do Mato Grosso do Sul, Campo Grande 79070-900, MS, Brazil; ligia.macedo@ufms.br

**Keywords:** lectin, trypsin inhibitor, rutin, vitexin

## Abstract

*Moringa oleifera* Lam. (horseradish tree) leaves demonstrate high nutritional value, are rich in proteins, and are widely used in folk medicine and food. This study investigated the presence of secondary metabolites and antinutritional proteins in leaf extract (LE) and the protein-rich fraction (PRF) derived from *M. oleifera* leaves, as well as the cytotoxicity to human cells, hemolytic activity, and in vivo acute toxicity and genotoxicity in mice. The flavonoids rutin and vitexin as well as trypsin inhibitors and lectins were detected in LE and PRF. Neither sample demonstrated toxicity against human peripheral blood mononuclear cells and both showed low hemolytic action. In vivo, LE and PRF did not show antinutritional effects and caused no death. The hematological parameters of the animals in the treated group were similar to those of the control. A significant increase in the serum levels of alanine aminotransferase and a discrete leukocyte infiltration with cytoplasmic vacuolization of the hepatocytes in the liver were detected in LE-treated animals. The preparations were not genotoxic or mutagenic. This study shows that LE and PRF are not antinutritional agents and presented low acute toxicity and no genotoxicity or mutagenicity. The present study contributes to the determination of the safety of using *M. oleifera* leaf proteins.

## 1. Introduction

The treatment of diseases using medicinal plants is an important practice in society. However, owing to their natural origin, plants are often used without scientific proof of efficacy and safety, and this may lead to the exertion of harmful effects depending on the dose and form of administration [1,2]. In vivo assays are necessary for the assessment of the safety of natural compounds, and toxicity is detected when animal death, changes in behavior, the presence of lesions in organs, and remarkable changes in serum biomarkers are observed [3]. Acute toxicity tests may help define the degree of intrinsic toxicity of a substance, aid in the identification of organs that may be the target of undesired effects, and may help in the selection of doses to be used for long-term studies [4]. 

*Moringa oleifera* Lam. (Moringaceae) is a species native to Asia and is popularly known as the drumstick tree or moringa. It is a perennial and fast-growing tree that can be found in tropical and semi-arid regions [5,6]. People use *M. oleifera* leaves to prepare salads and tea, and the flour derived from moringa leaf powder is consumed as a multivitamin supplement [7]. *M. oleifera* leaves demonstrate high nutritional value because of the presence of high levels of proteins, vitamins, potassium, calcium, phosphorus, iron, essential amino acids, antioxidants (including β-carotene), and flavonoids [8,9,10,11]. In traditional medicine, moringa leaves are used to treat sores, headaches, piles, fevers, sore throat, bronchitis, eye and ear infections, scurvy, and catarrh [12].

Reports describe that dry leaf powder consumption leads to a significant reduction in blood glucose levels in diabetic patients [13]. The use of a leaf methanolic extract resulted in a significant reduction in weight loss, polydipsia, glucose tolerance, and plasma levels of liver enzymes in rats with diabetes induced by streptozotocin [14]. Berkovich et al. [15] and Tiloke et al. [16] showed that aqueous leaf extracts caused apoptosis in several types of cancer cells, with the suppression of pro-inflammatory signaling pathways and promotion of an antiproliferative environment. The bioactivities of *M. oleifera* leaves have been associated with the presence of flavonoids, tannins, glucosinolates, carotenoids, and proteins [17]. 

*M. oleifera* leaves contain a high protein content (28.7%) with low in vitro digestibility, and the presence of trypsin inhibitors and lectins has been reported [18,19,20]. Lectins and trypsin inhibitors can be considered antinutritional factors that interfere with the absorption of nutrients and can even lead to pancreatic hypertrophy [21]. Alatorre-Cruz et al. [22] reported that a lectin fraction obtained from *Phaseolus acutifolius* A. Gray seeds exhibited toxicity in rats at a daily oral dose of 50 mg/kg administered for six weeks. The fraction led to an increase in the granulocyte count in the blood, caused hypertrophy with vacuolations and trabecular enlargement in the pancreas, and triggered atrophy of the small intestine villi and colon crypt points, without normalization in a two-week recovery period after the completion of treatment. 

Asare et al. [23] demonstrated that water extract derived from *M. oleifera* leaves did not exhibit toxicity in rats at a dose of 3000 mg/kg; however, in a literature survey, we revealed the lack of availability of studies pertaining to the examination of toxicity of protein preparations derived from leaves. The present study was conducted to investigate saline leaf extract (LE) and the protein-rich fraction (PRF) from *M. oleifera* leaves for the presence of lectin, trypsin inhibitor and non-protein secondary metabolites, and to determine the cytotoxicity to human cells, hemolytic activity, and acute toxicity and genotoxicity in mice. 

## 2. Results

The use of 0.15 M NaCl for protein extraction resulted in the achievement of LE containing protein (4.4 mg/mL) and the detection of only trypsin inhibitor activity (55.38 U/mg). Aiming to solubilize lectin, 0.1 M citrate-phosphate pH 3.0 containing 0.15 M NaCl was used as an extraction solution. In fact, this extract showed trypsin inhibitor (54.5 U/mg) and lectin activity (specific hemagglutinating activity: 1.97) and was used for production of the protein-rich fraction. The PRF contained 6.4 mg/mL of protein and showed trypsin inhibitor (87.89 U/mg) and lectin (specific hemagglutinating activity: 2.5) activities. LE and PRF were then selected for further investigation because they differed in their protein composition, which would enable the identification of the contribution of each class to the biological properties. 

**Figure 1:**
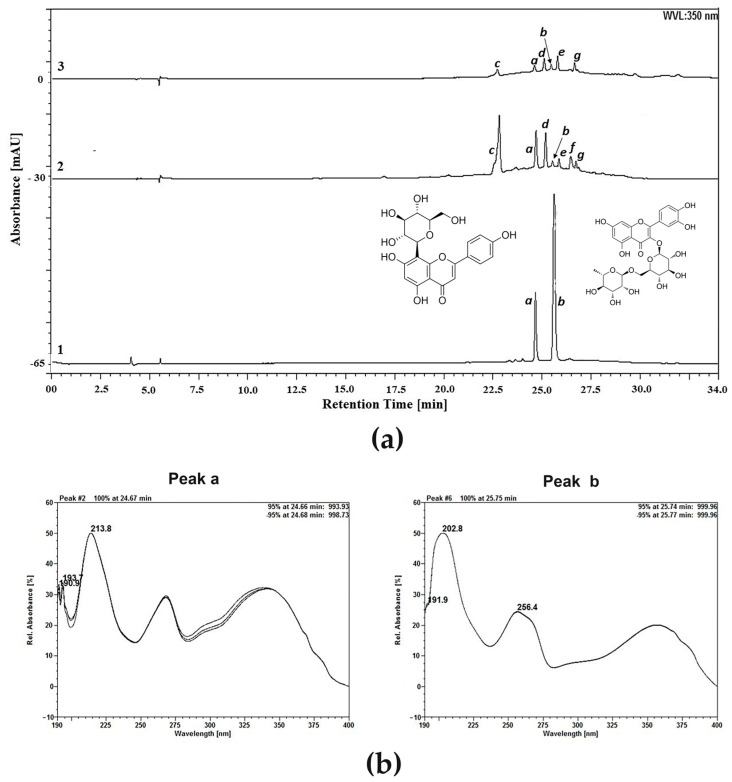
Chemical characterization of *Moringa oleifera* leaf extract (LE) and protein-rich fraction (PRF). (**a**) High-performance liquid chromatography (HPLC) profiles of vitexin and rutin (1), LE (2), and PRF (3) at a wavelength of 350 nm. The detected peaks are indicated by lower case letters (*a*–*g*). (**b**) Superimposed scanning spectra of the standards vitexin and rutin and their corresponding peaks (*a* and *b*, respectively) in the samples.

Thin-layer chromatography (TLC) analysis revealed the presence of flavonoids in the LE and PRF, according to the conditions and standards employed (see Section 4.4). High-performance liquid chromatography (HPLC) analysis was performed by monitoring the profile at a wavelength of 350 nm for the detection of flavonoids (Figure 1a). The presence of vitexin and rutin in both preparations was suggested in peaks with retention times around 24.70 min and 25.75 min, respectively. The scanning spectra of the standards and the peaks reinforced the indication of the presence of vitexin and rutin (Figure 1b). The levels of rutin for LE and PRF were 0.12 g% and 0.04 g%, respectively, while the vitexin content was 0.01 g% for LE and 0.05 g% for PRF. However, vitexin and rutin are not the single compounds in LE and PRF. According to the scanning spectra of the other peaks, they correspond to other flavonoid derivatives (Figure S1), but they did not match with any standard tested. Thus, they remain to be elucidated in the future using techniques such as liquid chromatography coupled to mass spectrometry (LC-MS).

LE and PRF did not exhibit cytotoxicity against human peripheral blood mononuclear cells (PBMCs) (Table 1) and caused a maximum hemolysis of 2.08% and 1.22%, respectively (Table 1), indicating low hemolytic action. In the assessment of LE and PRF for acute oral toxicity, mice treated with a dose of 2000 mg/kg were observed for 14 days and mortality was not observed. In relation to behavioral signs, stimulating signs were observed in the first 30 min in the group subjected to treatment with LE, such as self-cleaning and pedaling, followed by a depressive sign of decreased ambulation, in the interval of 30–45 min, when compared to the control. Only signs of decreased ambulation were observed in the animals treated with PRF during the period of hippocratic screening (60 min). These effects caused by LE and PRF ceased after 60 min. The water consumption and average body weight in animals treated with LE and PRF were similar to those in the control group (*p* > 0.05), while a decrease in feed consumption was detected in animals treated with LE (Table 2).

The determination of hematological and biochemical parameters is important to define the toxicity of a preparation because an alteration in such parameters indicates the ingestion of toxic compounds. The hematological parameters (Table 3) of the animals treated with LE and PRF were similar to those of the untreated group. Biochemical analysis (Table 4) revealed only a significant increase (*p* < 0.05) in alanine aminotransferase (ALT) levels in animals treated with LE.

Macroscopic analysis of the organs of mice treated with LE and PRF did not show changes in color, volume, or texture when compared to the control. Additionally, the relative weights of the heart, kidney, spleen, and liver did not differ significantly from the weights of the organs obtained from control animals (Table 5). 

**Figure 2:**
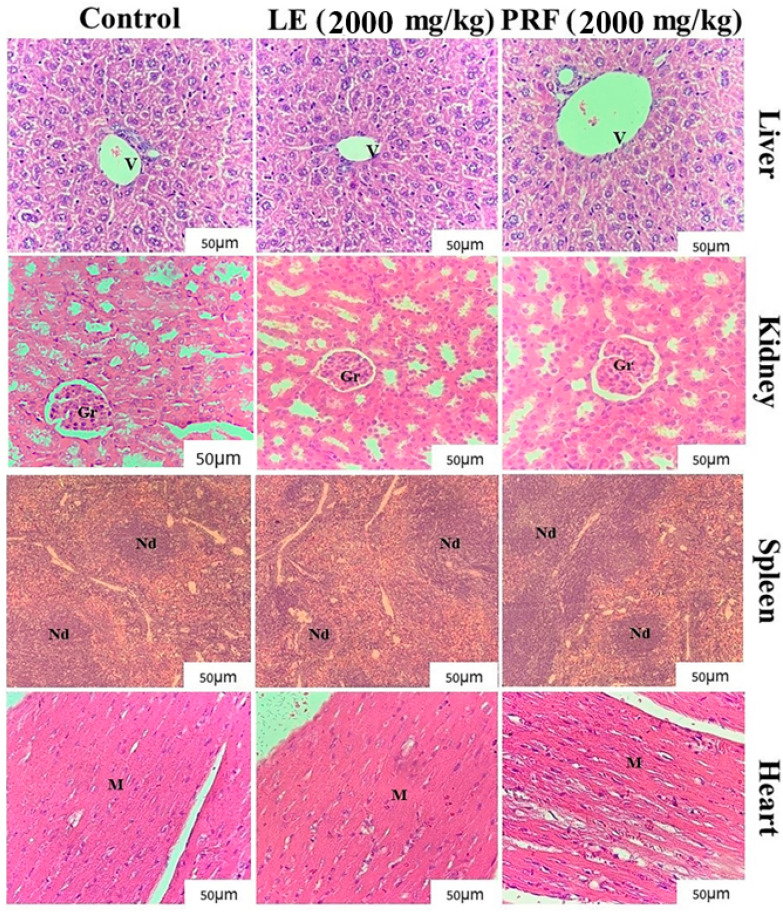
Representative photomicrographs of the liver, kidney, spleen, and heart of animals in the control groups (0.15 M NaCl), *M. oleifera* leaf extract (LE) (2000 mg/kg), and protein-rich fraction (PRF) (2000 mg/kg). Liver: Centrilobular veins (v) of different calibers, polygonal hepatocytes, and strands of regular hepatocytes can be observed in the images. Animals treated with LE presented with a slight leukocyte infiltrate in the presence of vacuolation in the cytoplasm of hepatocytes. Kidneys: well-defined structural components can be observed with a fibrous outer capsule, homogeneous glomeruli (Gr), and with the presence of the Bowman space in all treated animals. Spleen: the preservation of structures can be observed without changes in all treated animals. Heart: the myocardium (M) with sarcoplasm and integral fibers can be observed. Approximation: 400×.

Figure 2 shows the histological evaluation of the organs of mice derived from the control, LE, and PRF groups. The liver samples of animals treated with LE presented discrete leukocyte infiltrate with the presence of reversible-type vacuolization in the cytoplasm of hepatocytes, in agreement with the alterations found in ALT levels. In contrast, the livers of control animals and those treated with PRF presented homogeneous aspects with the presence of a thin external capsule and demonstrated the development of parenchyma, centrilobular veins of different calibers, polygonal hepatocytes, and strands of regular hepatocytes.

The histological findings obtained for the kidneys of animals treated with LE and PRF (Figure 2) were like those obtained for the animals in the control group, presenting with well-defined structural components with an external fibrous capsule, homogeneous glomeruli, and the presence of the Bowman space. Distorted proximal and distal tubules showed no changes in caliber or shape with the absence of inflammatory spots. Histological data were in accordance with those of the biochemical parameters of urea and creatinine and did not show significant changes in relation to the control group, indicating the absence of renal toxicity.

Figure 2 also depicts photomicrographs of the spleen and heart of the animals. The spleen architecture was preserved in animals treated with LE and PRF in the presence of white and red pulp, like the findings obtained for the control animals. The cardiac tissues of the control group and the groups treated with LE and PRF did not present myocardial degeneration with integrated sarcoplasm and fibers.

**Figure 3:**
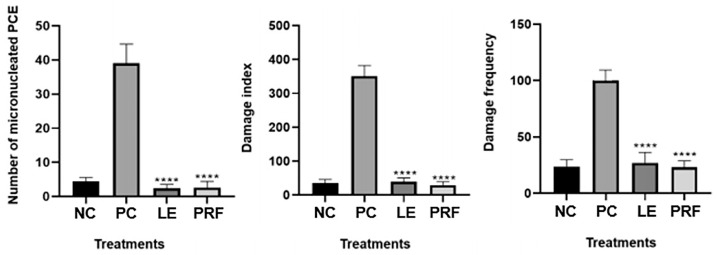
Micronucleus tests and comet assay. PCE: polychromatic erythrocytes; NC: negative control (saline); PC: positive control (cyclophosphamide dose: 25 mg/kg); LE (leaf extract) and PRF (protein-rich fraction) doses of 2000 mg/kg. The asterisks (****) represent statistically significant differences (*p* < 0.05).

The data obtained from the micronucleus and comet assays are shown in Figure 3. A comparison between the negative control and treated groups revealed that LE and PRF did not promote an increase in the frequency of micronuclei in polychromatic erythrocytes or an increase in the damage frequency (DF) or damage index (DI) of the nucleated blood cells. In the positive control group, there was an increase in the frequency of micronuclei, DF, and ID when compared to the negative control group. Our data indicated that LE and PRF at a dose of 2000 mg/kg did not pose genotoxicity or mutagenicity.

## 3. Discussion

Acute toxicity assays involving the oral administration of *M. oleifera* aqueous leaf extracts were conducted by Adedapo et al. [24] at doses ranging from 400 to 6400 mg/kg, by Awodele et al. [25] at doses ranging from 1000 to 3000 mg/kg, and by Asare et al. [23] with doses ranging from 400 to 2000 mg/kg. In all cases, no mortality was observed after treatment. Lectins and trypsin inhibitors belong to the group of possibly toxic plant proteins [26] but LE and PRF containing these protein classes do not promote mortality. Thus, it presented low toxicity (LD_50_ above 2000 mg/kg) according to protocol 423 of the OECD. 

Awodele et al. [25] also showed that the oral administration of water extract from *M. oleifera* leaves at doses of 400 to 6400 mg/kg promoted lethargy in the animals in the first two hours after administration. Similar behavioral changes were detected after LE and PRF treatments. Selvakumar et al. [27] showed that flavonoids (including rutin) possess the ability to cross the blood–brain barrier and demonstrate actions on the central nervous system, leading to the exertion of anxiolytic and sedative effects. Souza et al. [28] demonstrated that animals treated with hydroalcoholic extract of *Annona muricata* L. leaves containing rutin (5.20 mg/g), when administered (500 to 2000 mg/kg) once for 14 days, promoted effects such as sedation and decreased ambulation. The effects caused in mice by LE and PRF may be attributable to the presence of flavonoids. The rutin content in LE is three times more than that in PRF, and this may be related to the higher level of behavioral signs detected after LE treatment. Stimulating signals can also be attributed to stress caused during gavage. However, such a phenomenon might not cause the signs triggered by LE, since they were not detected after PRF administration via gavage. 

Unlike the findings reported in the present study, Adedapo et al. [24] showed that the use of 6.0% *M. oleifera* leaf water extract at a dose range of 400 to 2000 mg/kg promoted weight loss over a period of 21 days of treatment. The fact that LE and PRF did not reduce weight gain revealed that the trypsin and lectin inhibitors of *M. oleifera* leaves did not exert an antinutritional effect. Meenu-Krishnan and Murugan [29] did not detect behavioral changes or weight loss when animals were treated with a *Solanum aculeatissimum* Jacq. protease inhibitor (500 to 1000 g/kg) orally once for 7 days. 

On the other hand, a decrease in feed consumption was detected in animals treated with LE. Madingol et al. [30] reported that reduced intake could be attributed to the presence of toxic secondary metabolites, and the fact that PRF did not change this parameter suggested that the active constituent was a secondary metabolite and not a protein. Stress or physiological adaptation to the ingestion of new food can also reduce food consumption [31]; however, this hypothesis cannot explain fully the results, since PRF was administered under the same conditions as those considered for LE and did not change this parameter. Interestingly, the consumption of *M. oleifera* leaves has been considered by people for weight loss, and Nahar et al. [32] demonstrated the anti-obesity activity of *M. oleifera* leaf powder.

Only LE caused an increase in the ALT levels. Oliveira et al. [33] showed that animals that received oral (2000 mg/kg) ethanolic extract of *Morus alba* L. leaves containing flavonoid rutin presented an increase in ALT levels. The increase in ALT activity may be a result of the higher concentration of rutin in LE than that in PRF. Roy et al. [34] demonstrated that an orally administered vanadium–rutin complex (120 mg/kg) promoted the mortality of camundongos Balb/C and increased ALT levels, suggesting that the formation of a rutin–transition metal complex resulted in toxicity. However, in vitro and in vivo assays demonstrated that isolated flavonoids and extracts containing flavonoids usually demonstrate low toxicity and interesting biological properties from the perspective of human health [35]. Rutin did not exhibit cytotoxicity at a concentration of 200 µM used in rat hepatocytes, exerted a protective effect against thioacetamide-induced liver fibrosis, and prevented the development of methotrexate-induced hepatotoxicity in an animal model [36]. The histological data associated with the biochemical parameters revealed that LE and PRF did not cause severe liver damage, which matches the absence of mortality. 

The presence of some trypsin inhibitors and lectins can cause gastric inflammation, nausea, diarrhea, local bleeding, kidney damage, and destruction of the epithelium of the gastrointestinal tract [37,38]. However, the present study demonstrated that the presence of trypsin inhibitors and lectins in the PRF did not lead to histological damage. Patriota et al. [39] also showed that animals treated with trypsin inhibitor isolated from the flowers of *M. oleifera* (15 and 30 mg/kg) did not show changes in biochemical, hematological, and histological parameters. Ramos et al. [40] demonstrated that the lectin isolated from the leaves of *Schinus terebinthifolia* Raddi (1 and 5 mg/kg) was not toxic to animals treated orally for a period of 7 days, but animals treated with saline leaf extract (100 mg/kg) presented changes in the AST level and the number of peripheral blood monocytes. 

LE and PRF were not genotoxic or mutagenic agents. However, Asare et al. [23] detected genotoxicity after conducting treatment with a water extract derived from *M. oleifera* leaf at a dose of 3000 mg/kg. These genotoxic agents were probably not present in LE and PRF or are present at low concentrations.

## 4. Materials and Methods

### 4.1. Leaf Extract and Protein-Rich Fraction

After authorization from *the Instituto Chico Mendes de Conservação da Biodiversidade* (38,690) of the Brazilian Ministry of the Environment, leaves of *M. oleifera* Lam. were collected at the campus of *the Universidade Federal de Pernambuco* (UFPE) at Recife, Brazil (8°03′01.6″ S; 34°56′57.0″ W), in March 2018. A voucher specimen (number 73,345) was deposited at the herbarium Dárdano de Andrade Lima (*Instituto Agronômico de Pernambuco*, Recife, Brazil). 

Initially, the dry leaves were pulverized to obtain a powdered form of the sample. Dry powder (7.5 g) was homogenized with 0.15 M NaCl (100 mL) using a blender (5 min at 25 °C). Then, the mixture was filtered through filter paper and was centrifuged (9000× *g*, 15 min, 4 °C). The collected supernatant corresponded to leaf extract (LE). 

To obtain the protein-rich fraction (PRF), the leaf powder (7.5 g) was homogenized for 4 h at 25 °C in 0.1 M citrate-phosphate buffer pH 3.0, containing 0.15 M NaCl (100 mL) using a magnetic stirrer. The material obtained was filtered and centrifuged (9000× *g*, 15 min, 4 °C). The supernatant obtained therein was treated with ammonium sulfate at 60% saturation [41]. Thereafter, centrifugation (9000× *g*, 15 min, 4 °C) ensued and the precipitate was collected. Following this, it was dialyzed against distilled water, obtaining the PRF. 

### 4.2. Determination of Protein Concentration and the Presence of Trypsin Inhibitor and Lectin 

Protein amount was obtained using a standard curve (31.25–500 μg/mL) of bovine serum albumin (Sigma-Aldrich, St. Louis, MO, USA) [42]. Trypsin inhibitor amount was determined using 96-well microplates [43]. Samples (10 and 20 μL) were incubated with 5 μL of 0.1 mg/mL of bovine trypsin (Sigma-Aldrich) and 5 µL of 8 mM *N*-α-benzoyl-DL-arginyl-4-nitroanilide (BApNA), and the volume was adjusted to 200 µL using 0.1 M Tris-HCl pH 8.0 in 0.15 M NaCl. The sample was replaced with tris buffer for the control. The absorbance at 405 nm was determined at time zero and after incubation at 37 °C for 120 min; the assay was performed in triplicate. A unit of trypsin inhibitor activity is defined as the amount of inhibitor that decreased the absorbance by 0.01 compared to the control. 

The presence of lectins was evaluated in the hemagglutinating activity assay performed in 96-well V-bottomed microplates [44]. The sample (50 μL) was serially diluted in 0.15 M NaCl before the addition of 50 μL of a preparation (2.5%, *v*/*v*) of rabbit erythrocytes subjected to fixation with glutaraldehyde [45]. Hemagglutinating activity was recorded as the reciprocal of the highest dilution of the sample that promoted hemagglutination. Specific hemagglutinating activity is defined as the ratio between hemagglutinating activity (titer^−1^) and protein amount (mg/mL). 

### 4.3. Thin-Layer Chromatography (TLC)

Silica gel 60-F254 chromatographic plates (Macherey-Nagel, Düren, Germany) were analyzed in vats after saturation with the mobile phase for 15 min at 28 °C. LE, PRF, or standards (1 mg/mL) were applied manually. After elution, the plates were dried at 28 °C and observed under ultraviolet (254 and 365 nm) and visible light. Then, they were subjected to development using specific reagents for each metabolite. The following standards were used: hydrolysable tannins, gallic acid; condensed tannins, catechin; flavonoids, rutin, and quercetin; cinnamic derivatives, chlorogenic acid, and caffeic acid; terpenes and steroids, β-sitosterol; coumarins; saponins, escin; quinones, sennoside A; alkaloids, atropine; and reducing sugars, D-maltose.

### 4.4. High-Performance Liquid Chromatography (HPLC) Analysis

LE and PRF (5 mg) were placed in a 10 mL volumetric flask and subjected to solubilization in 50% (*v*/*v*) ethanol. Finally, they were filtered through PVDF membranes (0.45 µm). HPLC analysis was realized using the Ultimate 3000 HPLC system (Thermo Fisher Scientific, Waltham, MA, USA) aggregate to a photodiode array detector (DAD) and equipped with a binary pump (HPG-3×00RS, Thermo Fisher Scientific), a degasser, and an automatic sampler equipped with a 20 µL loop (ACC-3000, Thermo Fisher Scientific). The wavelength was set at 350 nm. Chromatographic separations were realized using the Phenomenex C18 column (250 mm × 4.6 mm i.d., 5 µm, Thermo Fisher Scientific) equipped with the Phenomenex C18 pre-column (4 mm × 3.9 µm, Thermo Fisher Scientific). The separations were performed at 25 °C. The mobile phases consisted of ultrapure water (A) or methanol (B) acidified with 0.05% trifluoroacetic acid, and the flow rate was adjusted to 0.7 mL/min. A gradient program was considered as follows: 0–5 min, 20–25% B; 5–10 min, 25–40% B; 10–20 min, 40–80% B; 20–25 min, 80–85% B; 25–30 min, 85–40% B; 30–32 min, 40–20% B; and 32–34 min, 20% B. Data were analyzed after triplicate injection and were processed using the Chromeleon 6.8 software (Thermo Fisher Scientific). The standards (vitexin and rutin; Sigma-Aldrich) prepared in methanol and diluted in purified water to 10 μg/mL were submitted for analysis under the same conditions described for the sample. The contents were calculated according to the linear equations derived from the standards used.

### 4.5. Cytotoxicity to Human Peripheral Blood Mononuclear Cells (PBMCs)

Blood samples (sample volume: 45 mL) were obtained from five healthy non-smoking donors who signed and provided an informed consent form. Blood samples were introduced in heparin tubes (Vacuette). PBMCs were obtained via density gradient centrifugation of the samples using Ficoll-Paque Plus (GE Healthcare Life Sciences, Uppsala, Sweden), and cell viability was checked using trypan blue (Sigma-Aldrich). The cells were used only when the viability was greater than 98%. PBMCs were cultured in the RPMI 1640 medium (Sigma-Aldrich) with 10% (*w*/*v*) fetal bovine serum (FBS; Sigma-Aldrich) in 24-well plates (TPP Techno Plastic Products, Trasadingen, Switzerland) at a density of 10^6^ cells/well.

The cells were cultured in the absence (control) or presence of LE or PRF (6.25–50.0 μg/mL) for 24 h. After centrifugation (450× *g*, 4 °C, 10 min), the supernatant was removed and 1.0 mL of phosphate-buffered saline (PBS) was added to the precipitate. The cells were re-suspended and subjected to centrifugation under the same conditions. The pellet was resuspended in 300 µL of binding buffer (10 mM HEPES pH 7.4, 150 mM NaCl, 5 mM KCl, 1 mM MgCl_2_, and 1.8 mM CaCl_2_) and transferred to a labeled cytometer tube. Annexin V (AnnV) conjugated to fluorescein isothiocyanate (FITC) (1:500) and propidium iodide (PI, 20 μg/mL) were added. Flow cytometry was performed using the FACSCalibur platform (BD Biosciences, San Jose, CA, USA), and the results were analyzed using the CellQuest Pro software 5.0.1 (BD Biosciences). AnnV–/PI+ cells were considered necrotic, and AnnV+/PI– cells were in the early stages of apoptosis. Double-negative cells were considered viable cells.

### 4.6. Hemolytic Assay

Blood samples (sample volume: 5–10 mL) were obtained from healthy volunteers and introduced in heparinized tubes. Human erythrocytes were isolated via centrifugation (500× *g*, 10 min). The erythrocytes were washed five times with 0.15 M NaCl. To each tube, 1.1 mL of erythrocyte suspension (2%, *v*/*v*) and 0.4 mL of LE or PRF (0.125–2.0 mg/mL of protein) were added. Amounts of 0.15 M NaCl and saponin (0.0025%) were used as negative and positive controls, respectively. After 60 min of incubation, centrifugation (1400× *g*, 28 °C, or 10 min) was performed and the absorbance of the supernatant at 540 nm was recorded. Hemolytic activity is expressed as a percentage of hemolysis compared to the positive control (100% hemolysis).

### 4.7. Animals

For the acute toxicity assay, Swiss female mice (*Mus musculus* L.; 30–35 g) were used; the animals were obtained from the bioterium of the *Instituto Keizo Asami* (iLIKA) of the UFPE. The mice were initially adapted for five days in the animal experimentation laboratory at the *Departamento de Bioquímica* of the UFPE and maintained at a temperature of 21 ± 1 °C with a 12:12 photoperiod and ad libitum access to food (Purin, São Paulo, Brazil) and water. 

### 4.8. Acute Oral Toxicity

#### 4.8.1. Treatments

LE and PRF were solubilized in 0.15 M NaCl, and acute toxicity was assessed via oral administration. The mice were allocated into the following three groups (*n* = 5): a control group (vehicle), and two experimental groups that received LE or PRF at a dose of 2000 mg/kg of body weight [46]. The mice were observed initially in the first 4 h and until the 14th day for depression or excitement signs [47]. Body weight and water and food consumption were evaluated daily. At the end of the 14th day of treatment, the mice were anesthetized using ketamine and xylazine solution (2:1, *v*/*v*, i.p.) for blood collection. Subsequently, the liver, kidney, spleen, and heart samples were collected for histological analysis. Additionally, the weight of each organ was determined, and the mass and relative weight were calculated (organ weight per 10 g of body weight).

#### 4.8.2. Biochemical and Hematological Analysis

Animal blood samples were obtained in a tube containing a separator gel. Serum levels of total protein, albumin, alanine aminotransferase (ALT), aspartate aminotransferase (AST), alkaline phosphatase (ALP), gamma-glutamyl transferase (GGT), urea, and creatinine were measured using specific kits (Labtest Diagnóstica, Lagoa Santa, Brazil). Hematological analysis was performed using blood samples obtained in tubes containing the anticoagulant ethylenediamine tetraacetic acid (EDTA) and were evaluated using an automatic analyzer (Animal Blood Counter-ABC Vet, Montpellier, France) and via optical microscopy considering the following parameters: erythrocyte count, hemoglobin level, hematocrit, mean corpuscular volume (MCV), mean corpuscular hemoglobin (MCH), mean corpuscular hemoglobin concentration (MCHC), and total and differentiated leukocyte counts.

#### 4.8.3. Histopathological Evaluation

Fragments of liver, kidney, spleen, and heart samples of the control and treated animals were subjected to fixation in buffered formalin (10%, *v*/*v*), gradually dehydrated using ethanol (70–100%), diaphanized in xylazine, and embedded in paraffin. Histological sections (5 mm) were subjected to staining with hematoxylin–eosin and mounted on cover slips. The sections were observed under an Olympus microscope coupled to a Moticam 1.3 MP digital camera (Motic Incorporation Ltd., Causeway Bay, Hong Kong).

### 4.9. Genotoxicity Assays

#### 4.9.1. Experimental Groups and Blood Collection

To assess the presence of genetic damage and mutagenesis, twenty male mice were randomly divided into four experimental groups (*n* = 5). The negative control (NC group) received 0.3 mL of 0.15 M NaCl, while the test groups received 2000 mg/kg of LE (LE group) and 2000 mg/kg of PRF (PRF group). NC, LE, and PRF were treated by gavage. For the positive control (PC group), animals received 25 mg/kg of cyclophosphamide (Sigma-Aldrich, C0768) intraperitoneally. Then, 6 h and 24 h after administration of the substance to perform the comet and micronucleus assays, respectively, blood samples were collected by incision at the tip of the syrup. Subsequently, the animals were sacrificed by administering high doses of anesthetics (ketamine 300 mg/kg and xylazine 30 mg/kg).

#### 4.9.2. Comet Assay

A blood sample of 15 μL obtained from the control and test groups was homogenized using 100 μL of low-melting-point agarose (0.5% LM), and this mixture was then deposited on slides coated with standard 0.5% agarose. Slides were covered with a coverslip and were incubated at 4 °C for 10 min. After cooling, the coverslips were removed and the slides were placed in a lysis solution (2.5 M sodium chloride [NaCl], 100 mM EDTA], 10 mM Tris, 1% Triton X-100, DMSO 10%, pH 13) for 48 h. After lysis, the slides were placed in the electrophoresis vessel and incubated with 1 M NaOH and 200 mM EDTA, pH 13.0, for 20 min and subjected to horizontal electrophoresis for 20 min at 300 mA and 32 V. Slides were subjected to neutralization for 15 min in 0.4 M Tris-HCl (pH 7.5), added to absolute ethanol for 5 min, and stained with 30 μL of ethidium bromide solution (0.0002% *w*/*v*) to visualize the nucleoids. Analyses were carried out under a fluorescence microscope (Zeiss-imager M2; Carl Zeiss AG, Jena, Germany) using a 40× objective and AlexaFluor 546 filter (Carl Zeiss AG, Jena, Germany). All procedures, including blood collection, were carried out under red light. One hundred nucleoids from each animal were evaluated and classified considering the proportion of DNA in the tail and head of the comet according to Collins et al. [48], and the damage index (DI) and damage frequency (DF) were calculated [49].

#### 4.9.3. Micronucleus Test

Blood samples (5 μL) from all treated animals were disposed on slides previously coated with acridine orange and covered with a cover slip [50]. Three slides were prepared per animal, and 2000 polychromatic erythrocytes (PCEs) were analyzed for the presence of micronuclei. The analysis was performed using a fluorescence microscope (Zeiss-imager M2) with a 40× magnification objective and AlexaFluor 488 filter (Carl Zeiss AG, Jena, Germany) [51].

### 4.10. Statistical Analysis

The data are expressed as mean ± standard deviation (SD) and were subjected to one-way analysis of variance (ANOVA) followed by Bonferroni’s test. Statistical significance was set at *p* < 0.05.

## 5. Conclusions

The extract (LE) and protein concentrate (PRF) derived from *M. oleifera* leaves containing flavonoids, lectin, and trypsin inhibitors did not exhibit cytotoxic, hemolytic, genotoxic, or mutagenic properties. The change in behavior and increase in ALT levels detected in vivo were probably due to the high dose administered. *M. oleifera* leaves are used for consumption as food and folk medicine, and this study shows that LE and PRF did not show an antinutritional effect and caused no death. LE and PRF are not genotoxic or mutagenic agents. The present study contributes to the determination of the safety of using *M. oleifera* leaf proteins and encourages continuity in the conduction of toxicological surveys.

## Figures and Tables

**Table 1:** Evaluation of cytotoxicity to human peripheral blood mononuclear cells (PBMCs) and hemolytic activity of LE and PRF from *Moringa oleifera* leaf.

Cytotoxicity Evaluation		Hemolytic Activity	
Concentration (µg/mL)	PBMC Viability (%)	Concentration (mg/mL)	Hemolysis (%)
**LE**		**LE**	
6.25	97.4 ± 0.16 a	0.12	0.00 ± 0.00 a
12.50	96.5 ± 0.31 a	0.25	0.00 ± 0.00 a
25.00	98.0 ± 0.08 a	0.50	0.30 ± 0.02 b
50.00	97.3 ± 0.18 a	1.00	0.78 ± 0.09 c
100.00	97.0 ± 0.30 a	2.00	2.08 ± 0.15 d
**PRF**		**PRF**	
6.25	94.4 ± 1.8 a	0.12	0.00 ± 0.00 a
12.50	96.7 ± 0.7 a	0.25	0.00 ± 0.00 a
25.00	97.1 ± 0.2 a	0.50	0.00 ± 0.00 a
50.00	96.0 ± 1.3 a	1.00	0.46 ± 0.11 b
100.00	95.8 ± 0.9 a	2.00	1.22 ± 0.02 e
**Negative control**	96.6 ± 1.0 a	**Negative control**	0.51 ± 0.14 b

Different lowercase (cytotoxicity) or uppercase (hemolysis) letters indicate significant differences (*p* > 0.05) between treatments.

**Table 2:** Water and food consumption of mice from control and treated with LE and PRF from *Moringa oleifera* leaves for 14 days.

Parameter	Control	LE	PRF
Water consumed (mL)	25.46 ± 0.80	23.55 ± 0.66	24.71 ± 0.33
Food consumed (g)	17.64 ± 1.21	13.14 ± 1.05 *	17.85 ± 1.65
Weight gain (g)	38.85 ± 3.09	40.01 ± 3.54	40.12 ± 3.47

Values represent the mean ± standard error of the mean (SEM) (*n* = 5/group). (*) Significant differences (*p* > 0.05) were found in comparison with control.

**Table 3:** Hematological parameters of mice treated with LE and PRF from *Moringa oleifera* leaf (2000 mg/kg per os) for 14 days.

Parameter	Control	LE	PRF
Erythrocytes (10^6^/mm^3^)	5.00 ± 0.12	4.99 ± 1.04	5.02 ± 0.32
Hematocrit (%)	38.85 ± 0.12	38.96 ± 0.35	39.07 ± 0.27
Hemoglobin (g/dL)	13.22 ± 0.11	13.22 ± 0.28	13.41 ± 0.20
MCV (fL)	84.00 ± 0.51	83.67 ± 0.61	84.21 ± 0.24
MCH (pg)	28.17 ± 0.32	28.20 ± 0.20	28.01 ± 0.35
MCHC (%)	35.22 ± 0.43	35.02 ± 0.34	35.47 ± 0.61
Leucocytes (10^3^/mm^3^)	6.26 ± 0.18	6.17 ± 0.42	6.28 ± 0.55
Segmented (%)	53.79 ± 0.66	54.74 ± 0.67	53.81 ± 0.79
Lymphocytes (%)	29.22 ± 0.32	30.08 ± 0.35	29.28 ± 0.40
Monocytes (%)	15.33 ± 0.55	15.09 ± 0.22	15.29 ± 0.42

Values represent the mean ± standard error of the mean (SEM) (*n* = 5/group). No significant differences (*p* > 0.05) were found in comparison with control.

**Table 4:** Biochemical parameters of blood of mice treated with LE and PRF from *Moringa oleifera* leaf (2000 mg/kg per os) for 14 days.

Parameter	Control	LE	PRF
Albumin (g/dL)	1.99 ± 0.09	2.10 ± 0.14	2.00 ± 0.11
ALT (U/L)	68.20 ± 0.22	72.95 ± 0.45 *	67.95 ± 0.45
AST (U/L)	92.09 ± 1.02	90.07 ± 0.93	91.07 ± 1.01
Total protein (g/dL)	6.20 ± 0.10	6.12 ± 0.22	6.19 ± 0.24
Alkaline phosphatase (IU/L)	12.61 ± 0.08	12.38 ± 0.24	12.43 ± 0.16
GGT (U/L)	9.60 ± 0.08	9.48 ± 0.38	9.54 ± 0.06
Creatinine (mg/dL)	0.23 ± 0.08	0.22 ± 0.07	0.20 ± 0.09
BUN	3.67 ± 0.12	3.52 ± 0.51	3.66 ± 0.14
Total Cholesterol (mg/dL)	78.45 ± 0.21	78.44 ± 0.76	79.00 ± 0.09
Triglycerides (mg/dL)	100.08 ± 0.91	101.32 ± 1.09	99.56 ± 0.75

ALT: alanine aminotransferase; AST: aspartate aminotransferase; GGT: gamma-glutamyl transferase; BUN: blood urea nitrogen. Values represent the mean ± SEM (*n* = 5/group). (*) Significant differences (*p* > 0.05) were found in comparison with control.

**Table 5:** Evaluation of the relative weight (g/10 g animal body weight) of organs from mice treated with LE and PRF from *Moringa oleifera* leaf (2000 mg/kg per os) for 14 days.

Parameter	Control	LE	PRF
Heart (g)	0.18 ± 0.02	0.18 ± 0.02	0.18 ± 0.00
Liver (g)	2.03 ± 0.14	2.50 ± 0.26	2.05 ± 0.22
Kidney (g)	0.57 ± 0.02	0.56 ± 0.04	0.58 ± 0.05
Spleen (g)	0.18 ± 0.00	0.19 ± 0.04	0.18 ± 0.01

Values represent the mean ± SEM (*n* = 5/group). No significant differences (*p* > 0.05) were found in comparison with control.

## Data Availability

Dataset available on request from the authors.

## Supplementary Materials

The following supporting information can be downloaded at: https://www.mdpi.com/article/10.3390/ph17081045/s1, Figure S1: Scanning spectra of the peaks *c*, *d*, *e*, *f* and *g* indicated in the HPLC profiles of *Moringa oleifera* leaf extract (LE) and protein-rich fraction (PRF) shown in Figure 1.
