# Correction: Alves et al. Evaluation of Cytotoxicity and Acute Oral Toxicity of Saline Extract and Protein-Rich Fraction from *Moringa oleifera* Lam. Leaves. *Pharmaceuticals* 2024, *17*, 1045

**DOI:** 10.3390/ph18040454

**Published:** 2025-03-24

**Authors:** Robson Raion de Vasconcelos Alves, Alisson Macário de Oliveira, Gabryella Borges dos Prazeres, Abdênego Rodrigues da Silva, Franciele Florencio Costa, Bárbara Rafaela da Silva Barros, Talita Giselly dos Santos Souza, Luana Cassandra Breintenbach Barroso Coelho, Cristiane Moutinho Lagos de Melo, Magda Rhayanny Assunção Ferreira, Luiz Alberto Lira Soares, Cristiano Aparecido Chagas, Maria Lígia Rodrigues Macedo, Thiago Henrique Napoleão, Mariana Pinheiro Fernandes, Patrícia Maria Guedes Paiva

**Affiliations:** 1 Departamento de Bioquímica, Centro de Biociências, Universidade Federal de Pernambuco, Recife 50670-901, PE, Brazil; robson.raion@ufpe.br (R.R.d.V.A.); alissonmacario@hotmail.com (A.M.d.O.); gabryella.borges@ufpe.br (G.B.d.P.); rodriguesabdenego@gmail.com (A.R.d.S.); lcbbcoelho@gmail.com (L.C.B.B.C.); thiago.napoleao@ufpe.br (T.H.N.); 2 Departamento de Farmácia, Centro de Ciências da Saúde, Universidade Federal de Pernambuco, Recife 50670-901, PE, Brazil; francieli.costa@ufpe.br (F.F.C.); magda.ferreira00@gmail.com (M.R.A.F.); luiz.albertosoares@ufpe.br (L.A.L.S.); 3 Departamento de Antibióticos, Centro de Biociências, Universidade Federal de Pernambuco, Recife 50670-901, PE, Brazil; barbara.sbarros@ufpe.br (B.R.d.S.B.); cristiane.melo@ufpe.br (C.M.L.d.M.); 4 Centro Acadêmico de Vitória, Universidade Federal de Pernambuco, Vitória de Santo Antão 55608-680, PE, Brazil; talitagiselly@hotmail.com (T.G.d.S.S.); cristiano.chagas@ufpe.br (C.A.C.); mariana.fernandes@ufpe.br (M.P.F.); 5 Departamento de Tecnologia de Alimentos e da Saúde, Faculdade de Ciências Farmacêuticas, Alimentos e Nutrição, Universidade Federal do Mato Grosso do Sul, Campo Grande 79070-900, MS, Brazil; ligia.macedo@ufms.br

## Figure Legend

In the original publication [1] there was a mistake in Figure 2 as published. The kidney image for control group is wrong and corresponded to the PRF group. The corrected Figure 2 showing the correct kidney image for control group appears below. The authors state that the description of the results in the text and the scientific conclusions are unaffected. This correction was approved by the Academic Editor. The original publication has also been updated.

## Figures and Tables

**Figure 2:**
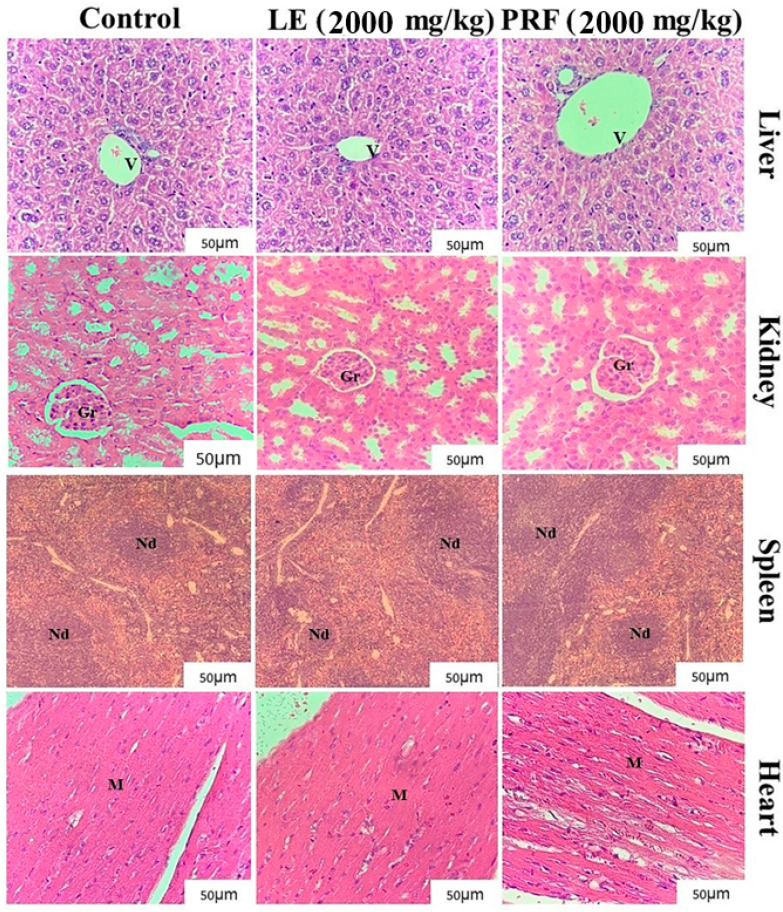
Representative photomicrographs of the liver, kidney, spleen, and heart of animals in the control groups (0.15 M NaCl), *M. oleifera* leaf extract (LE) (2000 mg/kg), and protein-rich fraction (PRF) (2000 mg/kg). Liver: Centrilobular veins (v) of different calibers, polygonal hepatocytes, and strands of regular hepatocytes can be observed in the images. Animals treated with LE presented with a slight leukocyte infiltrate in the presence of vacuolation in the cytoplasm of hepatocytes. Kidneys: well-defined structural components can be observed with a fibrous outer capsule, homogeneous glomeruli (Gr), and with the presence of the Bowman space in all treated animals. Spleen: the preservation of structures can be observed without changes in all treated animals. Heart: the myocardium (M) with sarcoplasm and integral fibers can be observed. Approximation: 400×.
