# Supplementary material for: Evaluation of Cytotoxicity and Acute Oral Toxicity of Saline Extract and Protein-Rich Fraction from Moringa oleifera Lam. Leaves
Source: Pharmaceuticals (Basel). 2024 Aug 8;17(8):1045. doi: 10.3390/ph17081045 (PMC11357182; doi:10.3390/ph17081045)
Supplement: Supplementary file 1 [file pharmaceuticals-17-01045-s001.zip › pharmaceuticals-3073273-supplementary.pdf]

Supplementary material

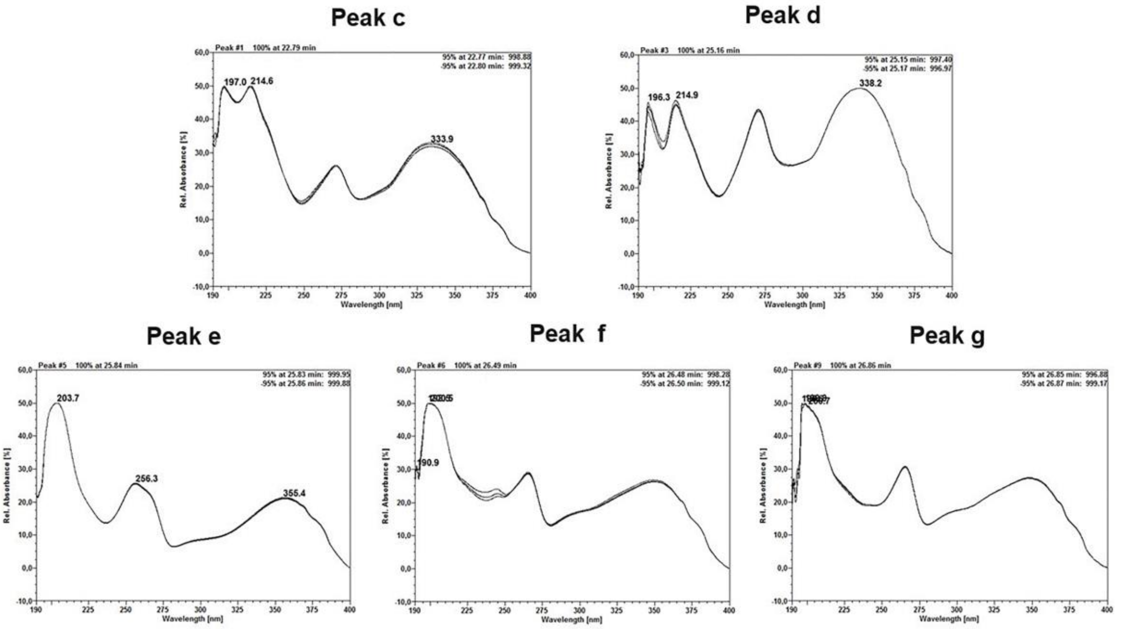

Figure S1. Scanning spectra of the peaks *c*, *d*, *e*, *f* and *g* indicated in the HPLC profiles of *Moringa oleifera* leaf extract (LE) and protein-rich fraction (PRF) shown in Figure 1.
